# An Integrin from Shrimp *Litopenaeus vannamei* Mediated Microbial Agglutination and Cell Proliferation

**DOI:** 10.1371/journal.pone.0040615

**Published:** 2012-07-09

**Authors:** Ying Zhang, Leilei Wang, Lingling Wang, Ning Wu, Zhi Zhou, Linsheng Song

**Affiliations:** 1 The Key Laboratory of Experimental Marine Biology, Institute of Oceanology, Chinese Academy of Sciences, Qingdao, China; 2 Research Center of Fishery Resources and Ecology, Shandong Marine Fisheries Research Institute, Yantai, China; 3 Graduate University of the Chinese Academy of Sciences, Beijing, China; New York University, United States of America

## Abstract

**Background:**

Integrins are a family of adhesion receptors which regulate cell proliferation, differentiation, leukocyte migration, and complement receptor-dependent phagocytosis. In invertebrates, as a cell adhesion receptor, β integrins play an important role for the balanced activation of immune defense responses especially during the encounter of infections. The present study attempts to characterize the immune functions of shrimp integrin (LvIntegrin) to have better understanding on the immune system and its regulation mechanisms in shrimps.

**Methodology:**

A shrimp integrin was identified from the Pacific white shrimp *Litopenaeus vannamei* (designated as LvIntegrin). Its full-length cDNA was of 2621 bp with an open reading frame (ORF) of 2439 bp encoding a polypeptide of 812 amino acids. The mRNA expression of LvIntegrin was significantly up-regulated at 3, 6 and 12 h after *Listonella anguillarum* challenge. The cDNA fragment encoding β integrin domains (βA and hybrid domain) of LvIntegrin was recombined and expressed in *Escherichia coli* BL21(DE3)-pLysS. The recombinant protein (rLvIntegrin) could significantly agglutinate the tested microbe including *E. coli* JM109, *L. anguillarum*, *Micrococcus luteus* and *Candida dattiladattila* in the presence of divalent cations. Moreover, when NIH3T3 cells were cultured with rLvIntegrin, the proliferation rate increased significantly in a dose-dependent manner.

**Conclusions:**

LvIntegrin, a shrimp β integrin was identified from *L. vannamei*, shared several highly conserved features. LvIntegrin exhibited broad-spectrum agglutination activity towards both bacteria and fungi and could improve the proliferation of NIH3T3 cells, indicating that LvIntegrin is involved in the immune response against microbe challenge and regulation of cell proliferation as a cell adhesion receptor in shrimp.

## Introduction

Many cell-mediated innate immune responses such as phagocytosis, nodulation and encapsulation are involved with multiple cell adhesion molecules on hemocytes surfaces [1]. These cell adhesion molecules are members of a limited number of gene families that include the integrins [2], immunoglobulin superfamily members, cadherins [3] and selectins [4]. Amongst these cell surface molecules, integrins appear as a versatile family of conserved αβ heterodimeric cell surface receptors, found in many diverse animal species, ranging from sponges to mammals [5]. There are 8 β and 18 α subunits found in vertebrates, composing a variety of at least 24 distinct integrins [6], which not only bind to extracellular molecules and generate intracellular signals regulating cell growth, survival, phagocytosis and migration events but also function as bi-directional signal transducer across the plasma membrane [7]. Till date, at least 17 β and 26 α invertebrate integrin subunits have been deposited in public database, and most of them were involved in early development [8]–[11].

The integrins of fruit fly and nematodes are the best characterized invertebrate integrins, and most of the studies are focused on their roles in embryonic development, muscle contraction, gut morphogenesis and linkage of epithelia cells to extracellular matrix and cuticle [12]. Recently, integrins have been proved to be involved in the innate immune responses in invertebrates. For instance, the integrins in *D. melanogaster* play a key role in regulating cell adhesion, migration, proliferation and apoptosis [11]–[14], and the β integrin domains from *Manduca sexta* and *Pseudoplusia includes* also participate in adhesion, encapsulation and phagocytosis of pathogen [2], [15], [16]. All the findings indicate that β integrins play important roles in the balanced activation of immune defense responses as a cell adhesion receptor during infectious encounter in invertebrates.

Shrimp is one of the most important commercial aquaculture species in the world, but shrimp aquaculture has been threatened by bacteria and virus diseases and suffered from huge economic losses in the past decades [17], [18]. Monitoring the host immune responses against pathogens would contribute to the development of management strategies for disease control and long-term sustainability of shrimp farming. β-integrin is of fundamental importance in innate immune responses as a cell adhesion receptor during the encounter of infections. Further characterization and more molecular information on β-integrins would be helpful for better understanding the immune system and its regulation mechanisms in invertebrates. In this context, the present study was done under the following objectives (1) to clone the full-length cDNA of a novel β-integrin from shrimp the Pacific white shrimp *Litopenaeus vannamei*, (2) to investigate its temporal response in hemocytes against *Listonella anguillarum* stimulation, (3) to characterize the microbial agglutinating and cell proliferation activities of recombinant LvIntegrin, and (4) to get further understanding of the immune system of *L. vannamei*.

## Results

### The Sequence Features and Phylogenetic Analysis of LvIntegrin cDNA

A 2621 bp nucleotide sequence representing the complete cDNA sequence of LvIntegrin was obtained by overlapping the fragments amplified with degenerate primers P1 and P2 and specific primers of P3–P6, oligo(dT)-adaptor and oligo(dG)-adaptor. The sequence was deposited in GenBank under accession number [GenBank: GU131148]. The complete sequence of LvIntegrin cDNA containing a 5′ untranslated region (UTR) of 64 bp, a 3′ UTR of 118 bp, and an open reading frame (ORF) of 2439 bp encoding a polypeptide of 812 amino acids with the predicted theoretical isoelectric point of 4.95. The N-terminus of LvIntegrin was a signal peptide as defined by Signal P program analysis, with a putative cleavage site located after position 18 (CEA-NIF). SMART analysis revealed that there are β integrin domains (N-terminal portion of extracellular region), three EGF-like repeats, integrin-β-tail domain, transmembrane segment and Cysteine-rich region of the C-terminus in LvIntegrin (Figure S1).

BLAST analysis revealed significant sequence similarity between LvIntegrin and other members of β integrins. It shared high similarity with the β-integrins from *Aedes aegypti* (70%), *Anopheles gambiae* (68%), *Bombyx mori* (65%), *Homo sapiens* (59%) and *Mus musculus* (58%). Several highly conserved features such as D^158^S^160^S^162^, D^165^D^167^K^168^ and SDL (K^198^- G^223^) were also identified in LvIntegrin by Multiple alignment of LvIntegrin with other members of β-integrin family (Figure S2). A phylogenetic tree was constructed based on the 23 amino acid sequences of β-integrin family members by the neighbor-joining method, and all the integrins were separated into two distinct groups (Figure S3). In the first group, LvIntegrin was clustered with other arthropod β-integrins and then formed an independent invertebrate group with β-integrins from mollusc *Crassostrea gigas* and *Biomphalaria glabrata*. In the second group, β-integrins from mammalian species clustered together, and then coalesced with those from poultry, amphibian and fish to form a vertebrate group (Figure S3).

**Figure 1:**
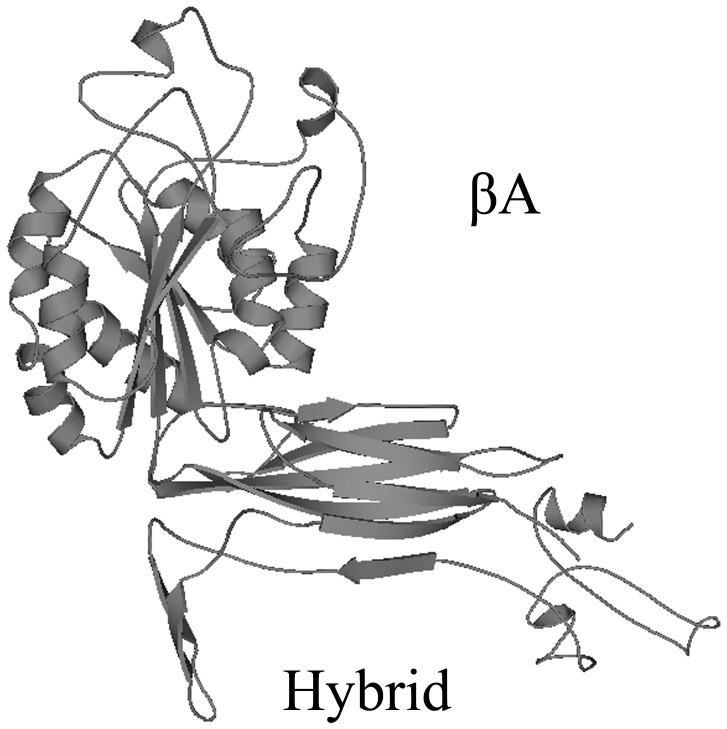
The predicted spatial β integrin domains, βA and Hybrid domain, in LvIntegrin as predicted by SWISS-MODEL.

Based on the similarity with other β integrin domains, the potential tertiary structure of β integrin domains (N-terminal portion of extracellular region) in LvIntegrin was established using the SWISS-MODEL prediction algorithm with the template [PDB:3ijeB], the structure of β-integrin from *H. sapiens*. There were two parts in the overall structure of β integrin domains, βA and Hybrid domain (Fig. 1). The βA domain was consisted of a central six β-strands sheet surrounded by eight helices, and a MIDAS motif occupied a crevice at the top of central β strand. The hybrid domain was similar to the I-set Ig domains.

### Temporal Change of LvIntegrin Transcripts after *L. anguillarum* Challenge

**Figure 2:**
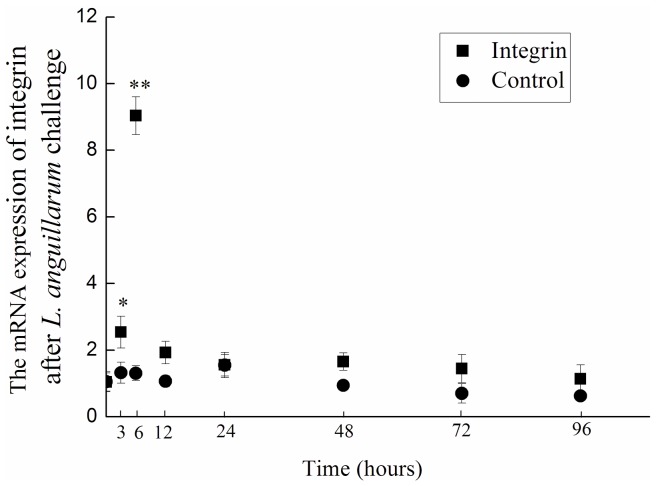
Real-time PCR analysis of LvIntegrin gene expression in hemocytes of shrimps challenged by *L. anguillarum* (white bars). Comparison of the level of LvIntegrin mRNA (relative to actin) among different time points was performed by Student’s t-test. Vertical bars represent the mean±S.E. (N = 6). Significant differences between challenged and blank group are indicated with an asterisk at *P*<0.05, and with two asterisks at *P*<0.01.

The LvIntegrin mRNA expression in hemocytes of shrimps after *L. anguillarum* was quantified by RT-PCR with β-actin as internal control. Its expression level was up-regulated at 3 h (2.7-fold of the blank, *P*<0.05) after *L. anguillarum* challenge, and reached the highest at 6 h which was 9.0-fold higher (*P*<0.01) than that of blank. And then the expression decreased to 2.0-fold (*P*<0.05) of the blank at 12 h post *L. anguillarum* challenge (Fig. 2).

### The Recombinant Protein of LvIntegrin

**Figure 3:**
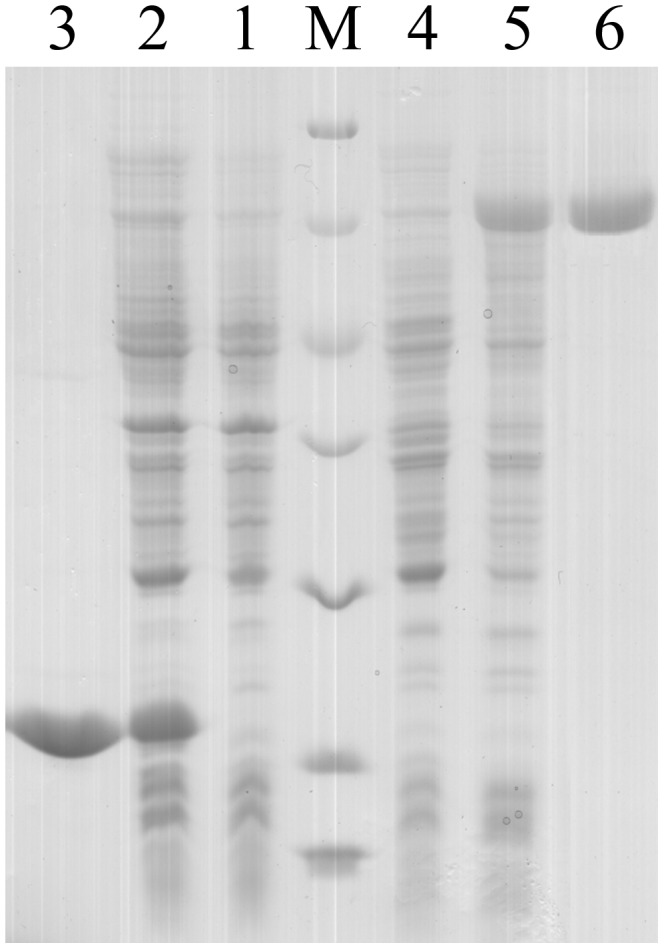
SDS-PAGE analysis of Trx and rLvIntegrin. Lane M: protein molecular weight standards 116.0, 66.2, 45.0, 35.0, 25.0, 18.4 and 14.4 (kDa); lane 1: negative control for Trx (without induction); lane 2: IPTG induced Trx; lane 3: purified Trx; lane 4: negative control for rLvIntegrin (without induction); lane 5: IPTG induced rLvIntegrin; lane 6: purified rLvIntegrin.

The recombinant plasmid pET-32a-LvIntegrin was transformed and expressed in *E. coli* BL21(DE3)-pLysS. After IPTG induction for 4 h, the whole cell lysate was analyzed by SDS-PAGE, and a distinct band with a molecular weight of 70 kDa was observed (Fig. 3), which was in accordance with the predicted molecular mass. After purification and refolding, the protein of rLvIntegrin showed the same molecular weight (70 kDa). Meanwhile, the transformant with pET-32a vector was induced and a unique 21 kDa expressed product representing Trx was detected and purified from the IPTG induced whole cell lysate (Fig. 3). The rLvIntegrin and rTrx protein was quantified to the concentration of 0.130 mg ml^−1^.

### The Microbe Agglutination Activity of rLvIntegrin

**Figure 4:**
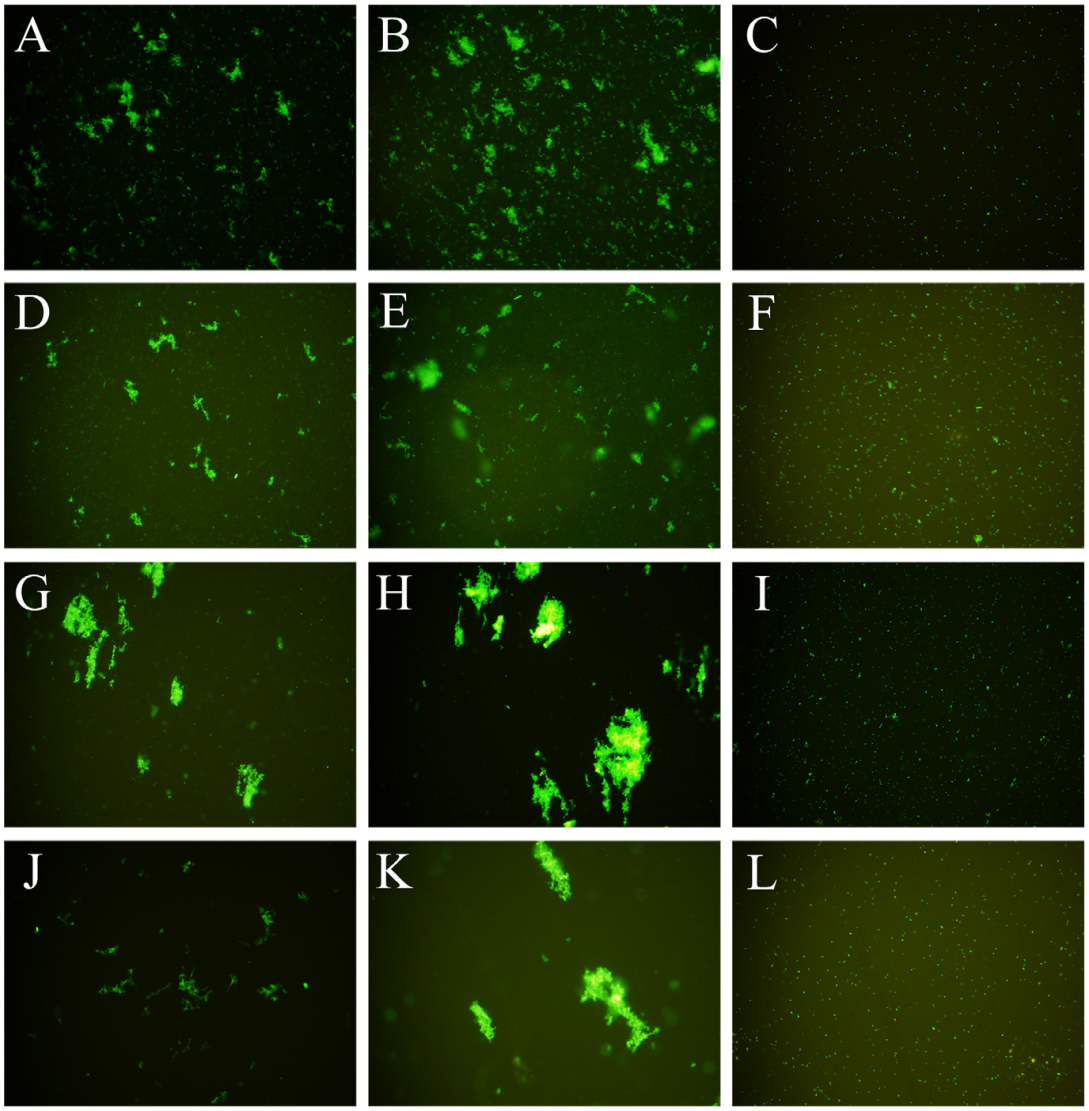
The bacterial and fungal agglutination induced by rLvIntegrin. rLvIntegrin labeled below was incubated with DAPI-stained *E. coli* JM109 (A) in Ca^2+^ buffer, (B) in the co-existence of Ca^2+^, Mg^2+^ and Mn^2+^ buffer, (C) in buffer only without added divalent-cation; *L. anguillarum* (D) in Ca^2+^ buffer, (E) in the co-existence of Ca^2+^, Mg^2+^ and Mn^2+^ buffer, (F) in buffer only without added divalent-cation; *M. luteus* (G) in Ca^2+^ buffer, (H) in the co-existence of Ca^2+^, Mg^2+^ and Mn^2+^ buffer, (I) in buffer only without added divalent-cation; *C. dattila dattila* (J) in Ca^2+^ buffer, (K) in the co-existence of Ca^2+^, Mg^2+^ and Mn^2+^ buffer, (L) in buffer only without added divalent-cation. The concentrations of Ca^2+^, Mg^2+^ and Mn^2+^ are 1 mmol L^−1^, 5 mmol L^−1^ and 0.4 mmol L^−1^ respectively.

The DAPI-stained Gram-negative bacteria *E. coli* JM109, *L. anguillarum*, Gram-positive bacteria *M. luteus* and fungi *C. dattiladattila* were used to test the agglutination activities of rLvIntegrin, and there is no agglutination observed in the absence of bivalent cation (Fig. 4C, F, I and L). While in the presence of Ca^2+^, rLvIntegrin (0.130 mg ml^−1^) could significantly agglutinate *E. coli* JM109, *L. anguillarum*, *M. luteus* and *C. dattiladattila* (Fig. 4A, D, G and J). The agglutinations were significantly accelerated when *E. coli* JM109, *L. anguillarum*, *M. luteus* and *C. dattiladattila* were incubated with rLvIntegrin in TBS-Ca-Mg-Mn buffer (Fig. 4B, E, H and K). The agglutination was significantly inhibited when the bacteria and fungi was incubated with rLvIntegrin in TBS-Ca-EDTA or TBS-Ca-Mg-Mn-EDTA buffer.

### The Effect of rLvIntegrin on Cell Proliferation

**Figure 5:**
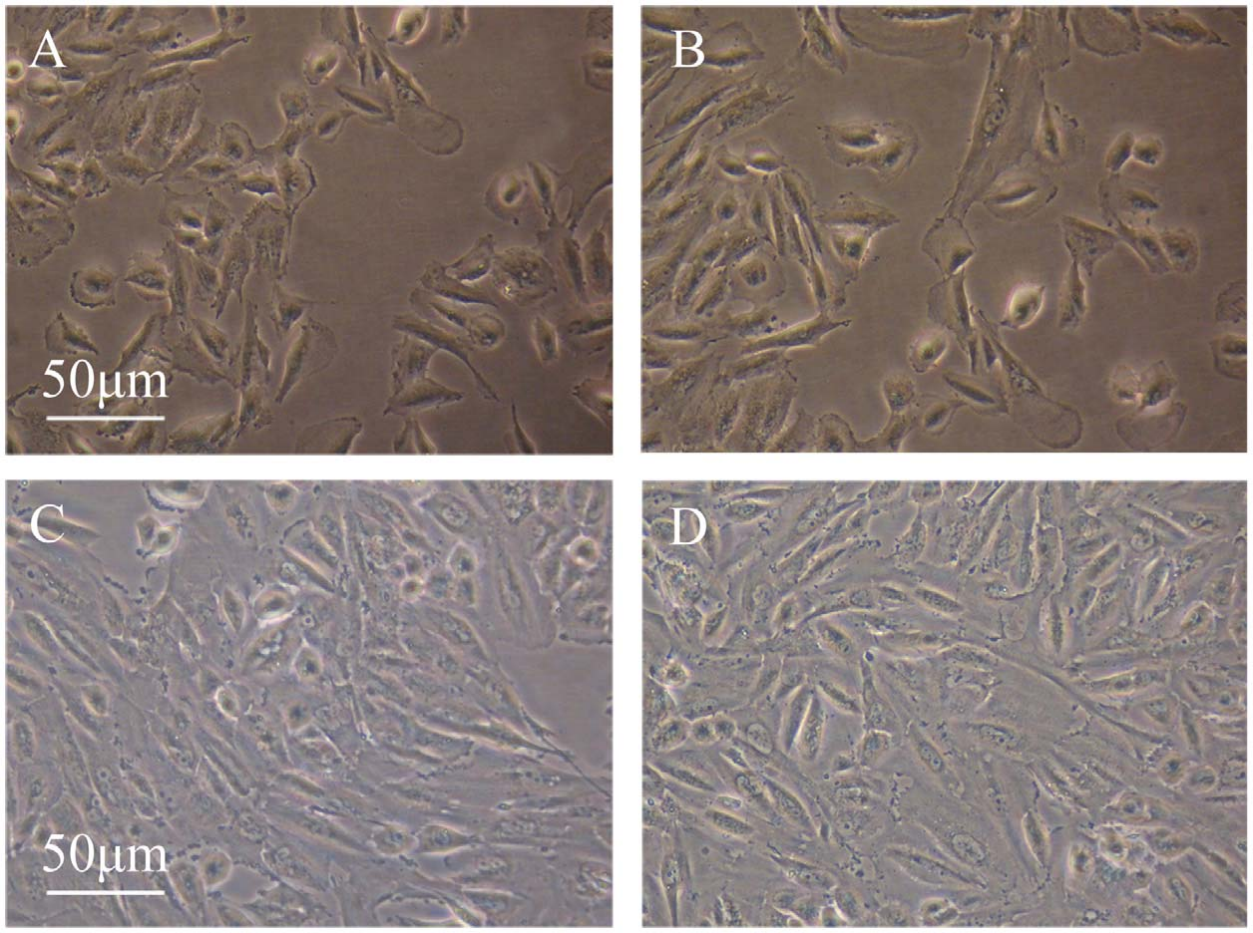
The NIH3T3 cells proliferation change expose to different concentration of rLvIntegrin. The NIH3T3 cells were incubated with (A) rTrx (0.130 mg/mL); (B) rLvIntegrin (0.013 mg/mL); (C) rLvIntegrin (0.065 mg/mL); (D) rLvIntegrin (0. 130 mg/mL).

**Figure 6:**
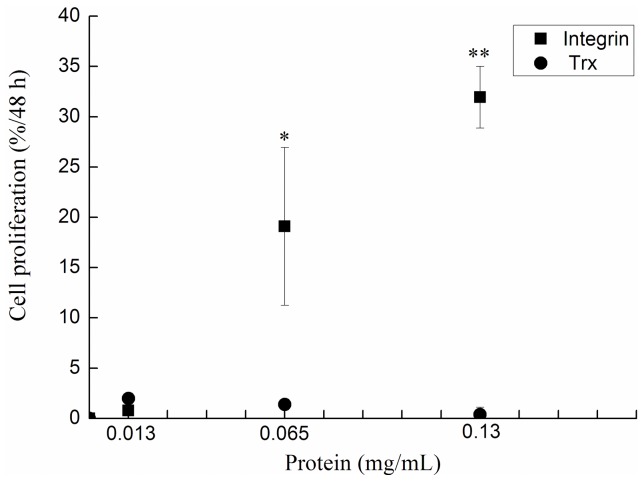
Influence of rLvIntegrin (0–0.130 **mg/mL) on NIH3T3 cells proliferation.** The cells were incubated with rLvIntegrin and rTrx in basal medium for 48 h. Cell numbers were determined by MTT assay. Results are normalized to untreated cells. All experiments were repeated six or more times.

MTT assay was performed to determine the effect of rLvIntegrin on the proliferation of NIH3T3 cells. The treatment with rLvIntegrin induced a significant improvement of NIH3T3 cell proliferation in a dose-dependent manner (Fig. 5 and Fig. 6). The cell proliferation rate of NIH3T3 cells treated with 0.130 and 0.065 mg ml^−1^ of rLvIntegrin increased, and it was almost 32-fold and 20-fold higher than that of cells treated with 0.130 and 0.065 mg ml^−1^ of rTrx, respectively. And no significant difference was found between 0.013 mg ml^−1^ of rLvIntegrin treatment group and rTrx control group.

## Discussion

Integrins are a large family of cell surface receptors that mediate cell-cell and cell-matrix interactions [19], and play critical roles in cell migration, differentiation and survival [7], [20]. In invertebrates, integrins have been identified from several species, and their involvement in early development was extensively studied [8]–[11]. However, the information and evidences on the involvement of integrins in invertebrate immune defence are still very limited. In the present study, a shrimp integrin was identified from *L. vannamei*, and its full-length cDNA contained an open reading frame (ORF) of 2621 bp encoding a polypeptide of 812 amino acids. The deduced amino acid sequence of LvIntegrin shared high similarities (58%–70%) with other β-integrins from arthropod and mammalian. Several highly conserved features, especially the MIDAS (D^158^S^160^S^162^, D^165^D^167^K^168^ and SDL (K^198^-G^223^)), were identified in the LvIntegrin. A phylogenetic tree was constructed based on the 23 amino acid sequences of β-integrin family members by the neighbor-joining method, and two distinct groups were separated clearly in the phylogenetic tree. In the first group, LvIntegrin was clustered with other arthropod β-integrin and then formed an independent invertebrate group with β-integrin from mollusc *Crassostrea gigas* and *Biomphalaria glabrata*. In the second group, β-integrin from mammalian firstly clustered together, and then got together with those from poultry, amphibian and fish to form a vertebrate group. Those structural characteristics and phylogenetic relationship indicated that LvIntegrin should be a new member of β-integrin family in shrimps.

The spatial structure of β integrin domains in LvIntegrin was established using the SWISS-MODEL prediction algorithm in base of the template 3ijeB [21]–[23], and it was similar to other known β integrin domains (Fig. 1). There were two parts in the overall structure of β integrin domains in LvIntegrin, βA and Hybrid domain. The βA domain was consisted of a central six β-strands sheet surrounded by eight helices, and a MIDAS motif occupied a crevice at the top of central strand. The tertiary and quaternary structural rearrangements in integrins can be triggered by its reaction with ligands, which are necessary for cell signaling. The structural rearrangements take place in βA [24], and βA is a major ligand-binding domain responsible for mediating protein-protein interactions. In the complex, βA acquires two cations, one of which contacts the ligand Asp directly and the other one stabilizes the ligand-binding surface [24]. The high affinity of cations to the MIDAS of βA mediates the interaction of βA with ligands [25]. The hybrid domain is similar to the I-set Ig domain and it contacts extensively with βA domain to display a mixed hydrophilic and hydrophobic nature [26]. The co-existence of βA and Hybrid domain in LvIntegrin indicates that LvIntegirn may bind to extracellular molecules and generates intracellular signals regulating immune responses in the presence of divalent cations.

In vertebrates, integrins shown to have the involvement in both developmental and immunological processes, such as early embryogenesis, regulation of cell proliferation and differentiation, leukocyte migration, and complement receptor-dependent phagocytosis [27]. However, the information about invertebrate integrins, especially their immunological roles, is still very far from well understood. Integrins or proteins with RGD-binding motifs in insects have been demonstrated to regulate adhesion, engulfment and phagocytosis [16], [28], [29]. In the present study, the expression of LvIntegrin in hemocytes was significantly up-regulated after *L. anguillarum* challenge, and the expression level was significantly higher than that of blank at 3, 6 and 12 h post challenge respectively, suggesting that LvIntegrin is an acute-phase protein having the significant roles to fight against the bacterial infection.

Cell adhesion is of fundamental importance to many biological processes, including inflammation and the immune responses [30]. Integrin-mediated cell adhesion to extracellular matrix components is central to the organization, maintenance, and repair of numerous tissues by providing anchorage and initiating signals that direct cell function [31], [32]. In order to further characterize LvIntegrin in terms of *in vitro* biological activities, the cDNA fragment encoding the mature peptide of β integrin domains in LvIntegrin was recombined into pET-32a+ plasmid and expressed in the host cell of *E. coli* BL21(DE3)-pLysS. In the presence of Ca^2+^, rLvIntegrin could significantly agglutinate all the tested bacteria and fungi, and bigger aggregations were observed in the co-existence of Ca^2+^, Mg^2+^ and Mn^2+^ (Fig. 4), suggesting that LvIntegrin has essential cell adhesion functions in shrimps. Nowadays the shrimps inhabited the aquatic environment with a large number of bacteria and fungi, are highly prone to severe diseases. Since there is no adaptive immunity in shrimps, the whole burden of anti-pathogen defence falls on the innate immune system [33], and cell adhesion acts as a first line of defence against pathogens. The integrins participate in binding and interactions of cells with the extracellular matrix, and mediate innate immune responses such as phagocytosis, nodulation and encapsulation [1]. As the LvIntegrin protein exhibited broad-spectrum agglutination activity towards both bacteria and fungi, we are encouraged to suggest that LvIntegrin has critical, individual roles in cell-substrate and cell-cell interactions during immune response and also serve essential cell adhesion functions in a divalent cation dependent manner.

Integrins also contribute to cell proliferation by providing a physical linkage between cytoskeletal structures and the extracellular matrix, and also participating in growth factor signaling through receptor tyrosine kinases (RTKs) [34]. In the present study, the rate of proliferation of NIH3T3 cells showed significant increase in a dose-dependent manner when they were treated with rLvIntegrin (Fig. 5 and Fig. 6). The cell proliferation rate of NIH3T3 cells treated with 0.130 and 0.065 mg ml^−1^ of rLvIntegrin increased, and it was almost 32-fold and 20-fold higher than that of cells treated with 0.130 and 0.065 mg ml^−1^ of rTrx, respectively. In vertebrates, β integrins transmit cell signaling to regulate cell proliferation as cell adhesion receptors. The results from the present study indicate that LvIntegrin could regulate cell proliferation and may have a major role in modulating the efficiency of proliferation factor signaling.

LvIntegrin, a shrimp β integrin was identified from L. vannamei, shared several highly conserved features, such as βA domain, hybrid domain and the metal ion-dependent adhesion site (MIDAS). To characterize the immune functions of LvIntegrin, the temporal mRNA expression of LvIntegrin in hemocytes of shrimps challenged with *L. anguillarum* and the bacterial and fungal agglutination activities of rLvIntegrin were determined. The expression of LvIntegrin in hemocytes was significantly up-regulated after *L. anguillarum* challenge, and in the presence of bivalent cation, rLvIntegrin could significantly agglutinate all the tested bacteria and fungi. The results suggested that LvIntegrin exhibited broad-spectrum agglutination activity towards both bacteria and fungi and served critical, individual roles in cell-substrate interactions during immune response. The rate of proliferation of NIH3T3 cells showed significant improvement in a dose-dependent manner when they were treated with rLvIntegrin, indicates that LvIntegrin would regulate cell proliferation. These results indicated that LvIntegrin was involved in the immune response against microbe challenge and regulation of cell proliferation as a cell adhesion receptor in shrimp *L. vannamei*.

## Materials and Methods

### Ethics Statement

The shrimps used in the present study are marine cultured, and all the experiments are conducted according to the regulations of local and central government. The experiments on this animal were approved by the Animal Care and Use Committee at Qingdao institute for the control of drug products with a permit number of SCXX (Shandong) 20090007, which complied with the National Institute of Health Guide for the Care and Use of Laboratory Animals.

### Animals, Immune Challenge and Hemocytes Collection

Healthy *L. vannamei* (body length 7–10 cm) were collected from a farm in Qingdao, China, and maintained in the filtered aerated seawater at 25±2°C for 1 week before processing. During the experiment, the shrimps were well-fed with commercial food, and water was totally exchanged daily. Eighty shrimps were kept in *L. anguillarum* (0.1×10^−8^ CFU mL^−1^)-contained seawater, and the untreated shrimps were employed as the control group. Six shrimps were randomly sampled at 3, 6, 12, 24, 48, 72 and 96 h post challenge. From each shrimp, about 1 mL hemolymph was collected into the tube containing equal volume of anticoagulant solution (115 mmol L^−1^ glucose, 336 mmol L^−1^ NaCl, 27 mmol L^−1^ sodium citrate, 9 mmol L^−1^ EDTA Na_2_·2H_2_O, pH 7.4). The haemolymph samples were centrifuged at 800×*g*, 4°C for 10 min to collect the hemocytes [35]. Total RNA was immediately extracted using Trizol reagent according to the manufacture’s protocol (Invitrogen).

### Cloning the Full-length cDNA of LvIntegrin

Two degenerate primers P1 and P2 (Table 1) were designed based on the sequences of arthropod integrin to clone a partial cDNA fragment of LvIntegrin. Two sense primers P3 and P4, and two anti-sense primers P5 and P6 (Table 1) were designed based on the sequences of this fragment to clone the full length cDNA of LvIntegrin by rapid amplification of cDNA ends (RACE). All the PCR programs were performed in a PTC-100 Programmable Thermal Controller Cycler (MJ Research), and the PCR products were cloned into the pMD18-T simple vector (TaKaRa) and sequenced in both directions with primers M-47 and M-RV (Table 1). The sequencing results were verified and subjected to cluster analysis.

**Table 1:** Names and sequences of primers used in this study.

Primer name	Sequence
**Lvintegrin**	
P1(forward)	5′- ARIYCTIAAGHTRHGRITSHAT-3′
P2(reverse)	5′- YTTCAGRTTRATCTGYGAAA-3′
P3(forward)	5′- AGATTAGGTGGCGGTCAGAAGC-3′
P4(forward)	5′- AGAGTGCCACCTCAATAACAAG-3′
P5(reverse)	5′- GTGGCATTTGTGGTGAGTGGAAGG-3′
P6(reverse)	5′- CACAAAGGAACCGAAGCCAAGT-3′
P7(forward)	5′- CCAGATTAGGTGGCGGTCAG-3′
P8(reverse)	5′- GTGCTGTTTGGCGACTTGATT-3′
**β-actin**	
AF(forward)	5′- CATCAAGGAGAAACTGTGCT-3′
AR(reverse)	5′- GATGGAGTTGTAGGTGGTCT-3′
oligo(dT)-adaptor	5′-GGCCACGCGTCGACTAGTAC(T)17-3′
oligo(dG)-adaptor	5′-GGCCACGCGTCGACTAGTAC(G)10-3′
**Sequencing primer**	
M-47(forward)	5′- CGCCAGGGTTTTCCCAGTCACGAC-3′
M-RV (reverse)	5′- GAGCGGATAACAATTTCACACAGG-3′

### Sequence Analysis

The cDNA sequence and deduced amino acid sequence of LvIntegrin were analyzed using the Blastp algorithm (Non-redundant protein sequences databases, all organisms, http://www.ncbi.nlm.nih.gov/blast) and the Expert Protein Analysis System (http://www.expasy.org/) respectively. The signal peptide was predicted with the SignalP 3.0 server (http://www.cbs.dtu.dk/service/SignalP), and the protein domains were revealed by the simple modular architecture research tool (SMART) version 4.0 (http://smart.embl-heidelberg.de/).

### Multiple Sequences Alignment, Tertiary Structures Prediction and Phylogenetic Analysis

The ClustalW Multiple Alignment program (http://www.ebi.ac.uk/clustalw/) was used to create the multiple sequence alignment of β integrin domains in LvIntegrin with other known β integrin domains from *A. gambiae*, *A. aegypti*, *B. mori*, *H. sapien* and *M. musculus*. The presumed tertiary structures were established for LvIntegrin using the SWISS-MODEL prediction algorithm (http://swissmodel.expasy.org/) and displayed by PyMOL version 0.97. A phylogenetic tree was constructed based on the sequence alignment by the neighbor-joining (NJ) algorithm using the Mega4.0 program. The reliability of the branching was tested by bootstrap re-sampling (1000 pseudo-replicates).

### The Temporal Expression of LvIntegrin mRNA in Hemocytes after *L. anguillarum* Challenge

The temporal mRNA expression of LvIntegrin in hemocytes of shrimps challenged with *L. anguillarum* was determined by quantitative real-time RT-PCR. Total RNA from hemocytes was extracted according to the protocol of Trizol (Invitrogen). M-MLV reverse transcriptase (Promega) was used to synthesize the single-strand cDNA with DNase I (Promega)-treated total RNA and oligo(dT)-adaptor primer (Table 1). The mixture was incubated at 42°C for 1 h, terminated by heating at 95°C for 5 min, and subsequently stored at –20°C.

The SYBR Green RT-PCR assay was carried out in an ABI PRISM 7300 Sequence Detection System. The amplifications were conducted in triplicates in a total volume of 50 µL. The thermal profile for quantitative RT-PCR program was 95°C for 5 min, followed by 40 cycles of 95°C for 5 s, 60°C for 31 s. The LvIntegrin specific primers P7 and P8 (Table 1) were used to amplify a fragment of 205 bp. Two β-actin primers, AF and AR (Table 1), were used to amplify a fragment of 214 bp as reference gene for internal standardization. Dissociation curve analysis of the amplification products was performed at the end of each PCR reaction to confirm that only one PCR product was amplified and detected.

After the PCR program, data were analyzed with SDS 2.0 software (Applied Biosystems). To maintain consistency, the baseline was set automatically by the software. The comparative average cycle threshold method was used to analyze the mRNA expression level of LvIntegrin. The C_T_ for the target amplified LvIntegrin gene and the C_T_ for the internal control β-actin was determined for each sample. Differences in the C_T_ for the target and the internal control, called ΔC_T_, were calculated to normalize the differences in the amount of total nucleic acid added to each reaction and the efficiency of RT-PCR. The blank was used as the reference sample, called the calibrator. The ΔC_T_ for each sample was subtracted from the ΔC_T_ of the calibrator; the difference was called ΔΔC_T_ value. The expression level of LvIntegrin gene could be calculated by 2^−ΔΔCT^, and the value stood for an n-fold difference relative to the calibrator [36]. All data were given in terms of relative mRNA expressed as mean ± S.E. (N = 6). The data were then subjected to one-way analysis of variance (one-way ANOVA). Difference was considered to be significant at *P*<0.05.

### Recombinant Expression of LvIntegrin and Protein Purification

The cDNA fragment encoding β integrin domains (βA and hybrid domain) of LvIntegrin was amplified with specific primers P17 and P18 (Table 1) with *Nco* I and *Sal* I sites at their 5′ end, respectively. The purified PCR products were inserted into pMD18-T simple vector, and digested with the restriction enzymes *Nco* I and *Sal* I. Then the PCR products were inserted into the *Nco* I/*Sal* I sites of pET-32a(+) plasmid to generate pET-32a-integrin recombinant which was sequenced by the primers of P17 and P18 to ensure in-frame insertion. The recombinant plasmid was transformed into *E. coli* BL21(DE3)-pLysS (Novagen). Positive clones were screened by PCR reaction with primers P17 and P18 and confirmed by nucleotide sequencing. The pET-32a vector without insert fragment was selected as a negative control, which could express a thioredoxin (Trx) with 6× His-tag in the prokaryotic expression system. Positive transformants and negative control were incubated in 100 ml SOB medium at 37°C with shaking at 220 rpm until the culture reached O.D. 600 of 0.6. Then 1 mmol L^−1^ isopropyl-β-D-thiogalactosidase (IPTG) was added to the medium under the same conditions. The recombinant LvIntegrin protein (rLvIntegrin) was purified by nickel affinity chromatography MagExtractor His-Tag NPK-700 (Toyobo) as described by the manufacturer. The resultant protein was separated electrophoretically on 15% SDS-polyacrylamide gel (SDS-PAGE) according to the method of Laemmli (1970) and visualized with Coomassie brilliant blue R250. The purified protein was refolded using a linear 6–0 M urea gradient in 50 mmol L^−1^ Tris-HCl, pH 7.4, 50 mmol L^−1^ NaCl, 10% glycerol, 1% glycine, 1 mmol L^−1^ EDTA, 0.2 mmol L^−1^ oxidized glutathione and 2 mmol L^−1^ reduced glutathione. The concentration of rLvIntegrin or rTrx protein was measured by BCA Protein.

### Bacterial and Fungal Agglutination Assay

The bacterial and fungal agglutination assay was performed as the method described by Zhang et al. [37]. Gram-negative bacteria *E. coli* JM109, *L. anguillarum*, Gram-positive bacteria *Micrococcus luteus* and fungal *Candida dattiladattila* were stained by 0.2 mg ml^−1^4′-6-Diamidino-2-phenylindole (DAPI) and then resuspended in TBS-Cabuffer (50 mmol L^−1 ^Tris-HCl, 50 mmol L^−1^ NaCl, 1 mmol L^−1^ CaCl_2_, pH 7.5) or TBS-Ca-Mg-Mnbuffer (50 mmol L^−1^ Tris-HCl, 50 mmol L^−1^ NaCl, 1 mmol L^−1^ CaCl_2_, 5 mmol L^−1^ MgCl_2_, 0.4 mmol L^−1^ MnCl_2_, pH 7.5) at 2.5×10^9^ cells ml^−1^ (for bacteria) or 2.5×10^8^ cells ml^−1^ (for fungi). A 10 µL bacteria or fungi suspension was added to 25 µL rLvIntegrin (final concentration of 0.13 mg ml^−1^ in buffer) or to 25 µL rTRX (0.13 mg ml^−1^) dissolved in the same buffer as negative control. The mixtures were incubated at room temperature for about 45 min and cells were then observed at the magnification of 400× under fluorescence microscopy (Carl Zeiss). To determine whether the agglutination of bacteria or fungi required Ca^2+^, Mg^2+^ and Mn^2+^, the bacteria and fungi stained by DAPI were incubated with rLvIntegrin (0.13 mg ml^−1^) in buffer containing 20 mmol L^−1^ EDTA under the same condition as described above.

### Cell Line Culture and Cell Proliferation Assay

NIH3T3 cell line was purchased from the American Type Culture Collection (ATCC, Rockville, MD), and cultured with DMEM (Invitrogen, NY) supplemented with 10% foetal bovine serum (Invitrogen, NY), and 100 IU/ml penicillin (Invitrogen, NY), 100 IU/ml streptomycin (Invitrogen, NY) in a humidified incubator at 37°C with 5% CO_2_. The NIH3T3 cells were plated in 96-well flat-bottomed tissue culture plates (5000 cells/100 µL/well). After 24 h incubation, cells were treated with rLvIntegrin and rTrx at various concentrations (0.013, 0.065 and 0.130 mg ml^−1^), and then incubated for another 48 h. After incubation, each treatment group cells were then observed under a microscope (Carl Zeiss), and then 10 µL of 3-(4, 5-dimethylthiazol-2-yl)-2, 5-diphenyltetrazolium bro-121 mide (MTT, Sigma) solution was added to each well for additional 4 h incubation. Two hundred micro liters of DMSO was added to each well and optical density (OD) was determined at 570 nm using a spectrophotometer (Bio-Tek Instruments, VT) [38].

## Supporting Information

Figure S1 **The domains by SMART analysis in LvIntegrin.** There were β integrin domains (N-terminal portion of extracellular region), three EGF-like repeats, integrin-β-tail domain, transmembrane segment and Cysteine-rich region of the C-terminus in LvIntegrin.
(TIF)

Figure S2 **Multiple sequence alignment by ClustalW of β integrin domains in LvIntegrin with other known β integrins.** Amino acid residues that are conserved in at least 60% of sequences are shaded in dark, and similar amino acids are shaded in grey. The species and the GenBank accession numbers are as follow: *L. vannamei* (**GU131148**), *Anopheles gambiae* (**CAC00630**), *Aedes aegypti* (**XP_001662592**), *Bombyx mori* (**NP_001161754**), *Homo sapiens* (**NP_002202**) and *Mus musculus* (**NP_034078**). The DXSXS and DDK amino acids in metal ion-dependent adhesion site (MIDAS) of LvIntegrin are marked with ▴, whereas SDL are bounded by double arrowhead. The crucial amino acids involved in recognizing the RGD ligands are marked with ♦.
(TIF)

Figure S3 **The phylogenetic tree based on the 23 amino acid sequences of β integrin family members by the neighbor-joining method.** The groups corresponding to vertebrate and invertebrate subfamilies, are marked with big brackets. The numbers at the forks indicate the bootstrap. The species and the GenBank accession numbers are as follow: *H. sapiens* (NP_002202), *Felis catus* (NP_001041625), *Ovis aries* (ABY71046), *Sus scrofa* (NP_999133), *Camelus dromedarius* (ACT68325), *Orytolagus cuniculus* (XP_002721235), *M. musculus* (NP_034708), *Ornithorhynchus anatinus* (XP_001508168), *Gallus gallus* (NP_001034343), *Xenopus laevis* (NP_001081286), *Xenopus tropicalis* (NP_989160), *Salmo salar* (ACN10531), *L. vannamei* (GU131148), *Tribolium castaneum* (XP_969214), *B. mori* (NP_001161754), *Spodoptera litura* (ACU32665), *Plutella xylostella* (ACS66819), *Acyrthosiphon pisum* (XP_001949887), *A. aegypti* (XP_001662592), *Culex quinquefasciatus* (XP_001848248), *A. gambiae* (CAC00630), *Crassostrea gigas* (BAB62173) and *Biomphalaria glabrata* (AAC67503).
(TIF)
