# Supplementary figures and images for: An Integrin from Shrimp Litopenaeus vannamei Mediated Microbial Agglutination and Cell Proliferation
Source: PLoS One. 2012 Jul 9;7(7):e40615. doi: 10.1371/journal.pone.0040615 (PMC3392225; doi:10.1371/journal.pone.0040615)

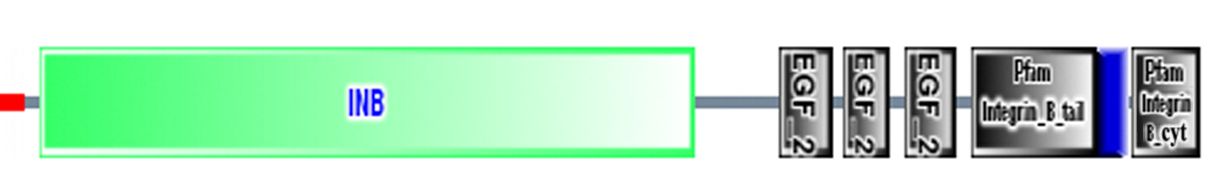

Supplement: Figure S1 — The domains by SMART analysis in LvIntegrin. There were β integrin domains (N-terminal portion of extracellular region), three EGF-like repeats, integrin-β-tail domain, transmembrane segment and Cysteine-rich region of the C-terminus in LvIntegrin. (TIF) [file pone.0040615.s001.tif]

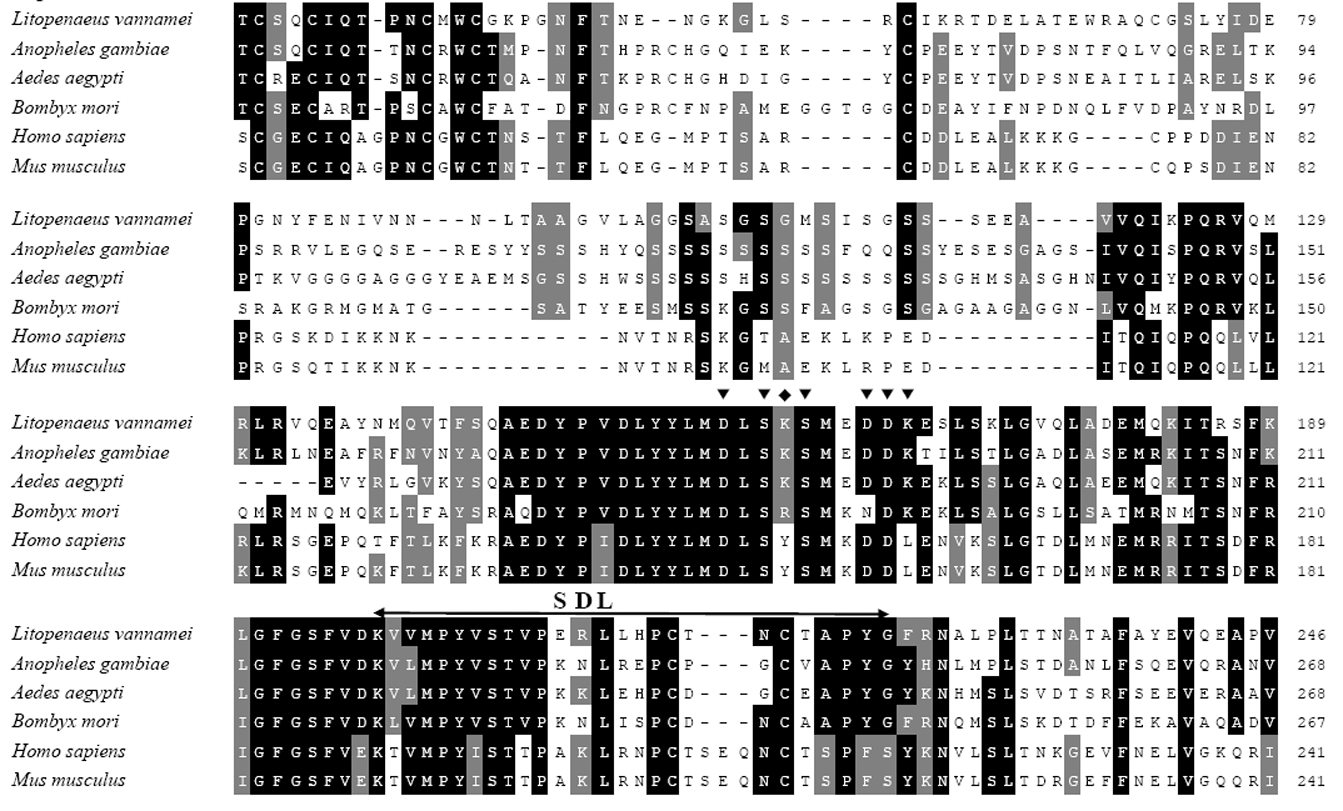

Supplement: Figure S2 — Multiple sequence alignment by ClustalW of β integrin domains in LvIntegrin with other known β integrins. Amino acid residues that are conserved in at least 60% of sequences are shaded in dark, and similar amino acids are shaded in grey. The species and the GenBank accession numbers are as follow: L. vannamei (GU131148), Anopheles gambiae (CAC00630), Aedes aegypti (XP_001662592), Bombyx mori (NP_001161754), Homo sapiens (NP_002202) and Mus musculus (NP_034078). The DXSXS and DDK amino acids in metal ion-dependent adhesion site (MIDAS) of LvIntegrin are marked with ▴, whereas SDL are bounded by double arrowhead. The crucial amino acids involved in recognizing the RGD ligands are marked with ♦. (TIF) [file pone.0040615.s002.tif]

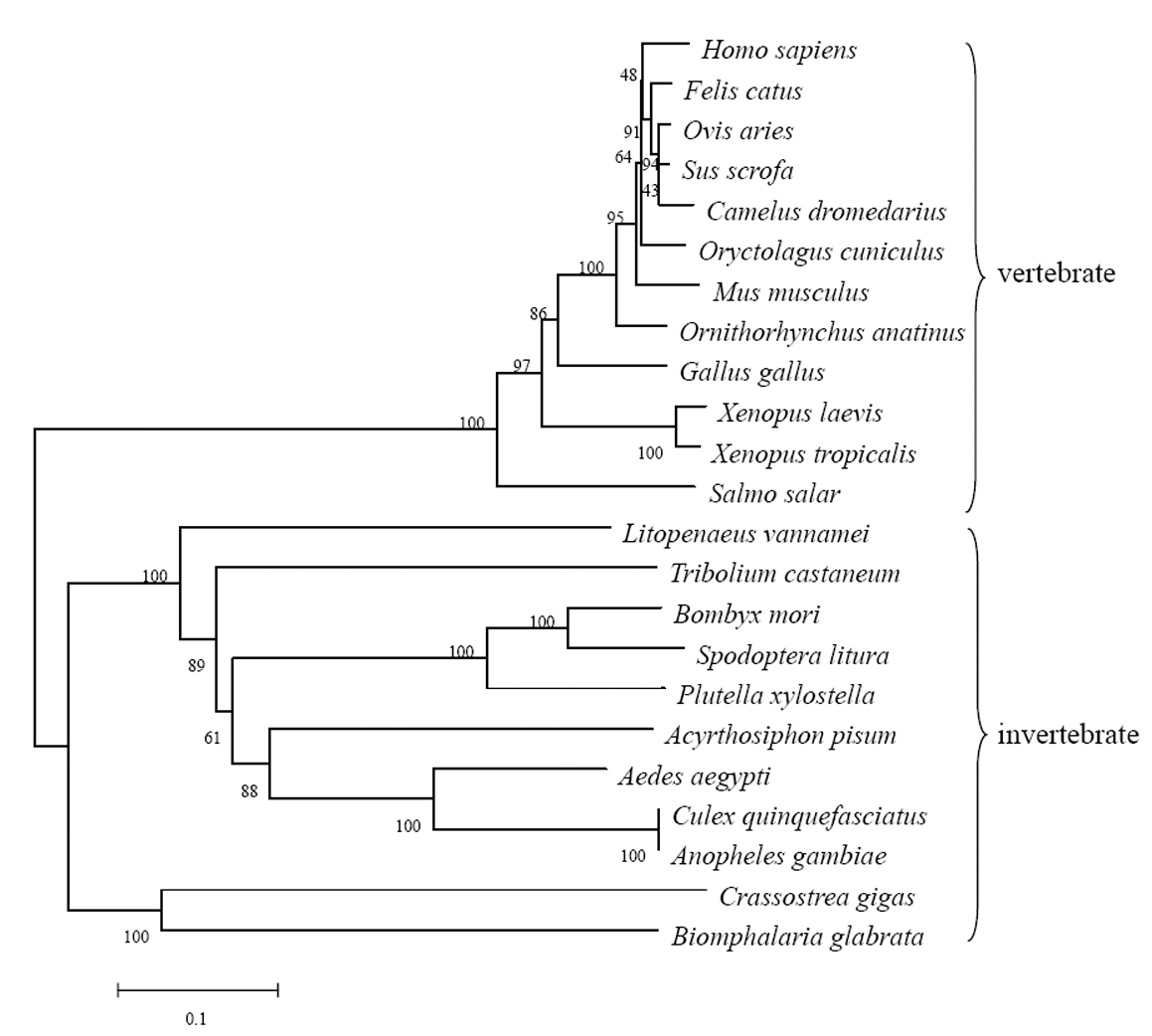

Supplement: Figure S3 — The phylogenetic tree based on the 23 amino acid sequences of β integrin family members by the neighbor-joining method. The groups corresponding to vertebrate and invertebrate subfamilies, are marked with big brackets. The numbers at the forks indicate the bootstrap. The species and the GenBank accession numbers are as follow: H. sapiens (NP_002202), Felis catus (NP_001041625), Ovis aries (ABY71046), Sus scrofa (NP_999133), Camelus dromedarius (ACT68325), Orytolagus cuniculus (XP_002721235), M. musculus (NP_034708), Ornithorhynchus anatinus (XP_001508168), Gallus gallus (NP_001034343), Xenopus laevis (NP_001081286), Xenopus tropicalis (NP_989160), Salmo salar (ACN10531), L. vannamei (GU131148), Tribolium castaneum (XP_969214), B. mori (NP_001161754), Spodoptera litura (ACU32665), Plutella xylostella (ACS66819), Acyrthosiphon pisum (XP_001949887), A. aegypti (XP_001662592), Culex quinquefasciatus (XP_001848248), A. gambiae (CAC00630), Crassostrea gigas (BAB62173) and Biomphalaria glabrata (AAC67503). (TIF) [file pone.0040615.s003.tif]
